# The distress thermometer as a predictor for survival in stage III lung cancer patients treated with chemotherapy

**DOI:** 10.18632/oncotarget.14151

**Published:** 2016-12-24

**Authors:** Mark de Mol, Brenda L. den Oudsten, Mieke Aarts, Joachim G.J.V. Aerts

**Affiliations:** ^1^ Department of Pulmonary Medicine, Erasmus University Medical Centre, Rotterdam, The Netherlands; ^2^ Department of Pulmonary Medicine, Amphia Hospital, Breda, The Netherlands; ^3^ Department of Medical and Clinical Psychology, Centre of Research on Psychological and Somatic Disorders (CoRPS), Tilburg University, Tilburg, The Netherlands; ^4^ Netherlands Comprehensive Cancer Organisation (IKNL), Utrecht, The Netherlands

**Keywords:** distress thermometer, health related quality of life, survival, cancer, chemotherapy

## Abstract

**Background:**

Depression and Health Related Quality of Life have been associated with prognosis in lung cancer. As the Distress Thermometer measures emotional problems and may share similarities with aspects of Health Related Quality of Life, we aimed to retrospectively assess the prognostic value of the Distress Thermometer in lung cancer patients treated with chemotherapy.

**Methods:**

Patients with stage III lung cancer who were treated at the day-care oncology unit with chemotherapy containing carboplatin from 2009 to 2014 and in whom a Distress Thermometer was performed at the time of the first cycle of chemotherapy were included in this study.

**Results:**

In total, one hundred and thirteen patients were included in the analysis. In the simple Cox regression analysis, overall survival did not significantly differ according to Distress Thermometer score. No significant differences in Distress Thermometer score according to stage, histology, (intended) treatment, age, sex, and comorbidity were observed. Also in a multivariable model the Distress Thermometer was not prognostic for overall survival, whereas sex and (intended) treatment was.

**Conclusions:**

In this study no prognostic value of the Distress Thermometer could be established in patients with stage III lung cancer treated with carboplatin. Further research is warranted to address this issue.

## INTRODUCTION

Distress reflects the spectrum of psychological problems (i.e. cognitive, emotional, social, and spiritual) associated with a diagnosis and treatment of cancer and can be measured by the Distress Thermometer (DT) [1–3]. In general, the DT is completed together with a problem list. The clinical application of the DT has been extensively investigated in patients with different forms and stages of malignancies, demonstrating acceptable to good accuracy in detecting distress [4–7] as well as change in distress [8]. One study in patients diagnosed with breast cancer demonstrated that moderate to severe distress was related to a significant decrease in Health Related Quality of Life (HRQoL) and that for the Quality of Life (QoL) scales for which a minimally important difference has been established this decrease ranged from two to three and a half times the established minimally important difference [9]. According to the results of this study, the DT could address aspects of distress beyond psychological problems and is therefore linked to HRQoL. Furthermore, the resemblance of items of the problem list with items of HRQoL questionnaires (e.g. the European Organization for Research and Treatment of cancer Quality of Life Questionnaire-Core 30 (EORTC QLQ-C30)), and the good to moderately strong relation of the problem list with the DT [6] support an association with HRQoL. To date, multiple studies have evaluated HRQoL as a predictor of survival in lung cancer patients [10–16]. Overall/global HRQoL is often observed to be a prognostic factor in these studies [10, 13–16]. Considering that depression has been associated with a decreased survival in patients with lung cancer [17, 18] and the considerable overlap between the problem list and HRQoL, the DT may be utilised as a fast, efficient, and promising tool to provide prognostic information similar as overall/global HRQoL does. Especially in lung cancer patients with a limited prognosis and who are at risk for cancer and treatment related adverse events and thus a decline in HRQoL this may be of importance. As the relation of the DT with survival has not been investigated before in lung cancer, we hypothesized that the DT is a predictor for overall survival (OS) after correction for age, gender, comorbidity, histology.

## MATERIALS AND METHODS

### Patient selection

Patients diagnosed with lung cancer treated at the day-care oncology unit of a large teaching hospital (Amphia Hospital, Breda, The Netherlands) specialized in lung cancer care from August 2009 until August 2014 were retrospectively enrolled in our study if they met the following criteria: they were aged 18 years or older, were diagnosed with stage III non-small cell lung carcinoma (NSCLC) or stage III small cell lung carcinoma (SCLC) according to TNM 7^th^ edition [19], were treated with first line chemotherapeutic regiments containing carboplatin, had a level of functioning which indicated that completion of the DT could be beneficial to optimize care, and had completed the DT at least at the time of the first cycle of treatment. We limited our inclusion to patients treated with carboplatin as the DT was more consistently performed in the day-care clinic of our department than at the clinical oncology unit. To optimize homogeneity of the patient sample only patients with stage III disease were analyzed as this was the largest population in our series. Patients with cisplatin treatment were not included as they require hospitalization. If no information on clinical treatment or survival was available, patients were excluded. As the included patients received standard care and were not exposed to additional interventions this study did not fall under the Medical Research Involving Human Subjects Act (WMO). In addition, informed consent of each patient was not required as the all data was handled to Dutch privacy law Therefore, permission of a medical ethics committee was not necessary.

### The Distress Thermometer

The DT is a visual analogue scale originally developed to describe the level of distress patients experience. Its scale ranges from 0 (*no distress*) to 10 (*extreme distress*) [1]. The DT is completed together with the problem list by patients at the time of the first, third, and fourth cycle of chemotherapy at our department. The Dutch version of the problem list comprises 47 items. It addresses practical, social, emotional, spiritual, and physical problems. The psychometric properties of the DT combined with the Dutch problem list have been investigated by Tuinman et al. [6]. They observed a good internal consistency, except for practical problems (α= 0.60) and spiritual problems (α= 0.64). In addition, a strong correlation between the DT and emotional problems (r= 0.61), physical problems (r= 0.64), and the total problem list (r= 0.68) was found. Tuinman et al. reported a sensitivity of 0.85 and a specificity of 0.69 of the DT at a cut-off value of five after performing receiver operator characteristics analysis with a Hospital Anxiety Depression Scale score of ≥ 15 as a gold standard [6].

### Additional information

Sociodemographic information (age, gender), comorbidity, histological tumor type (adenocarcinoma, squamous cell carcinoma, NSCLC otherwise not differentiated or adenocarcinoma in situ, and SCLC, cancer stage according to TNM 7^th^ edition (IIIA and IIIB; patients originally staged according to TNM 6^th^ edition were restaged using TNM 7^th^ edition) [19], treatment (chemotherapy, surgery in combination with (neo)adjuvant chemotherapy or chemotherapy in combination with radiotherapy (i.e. concurrent or sequential), and OS was retrieved from the electronic patient information system and the cancer registration of the Netherlands Comprehensive Cancer Organisation.

### Statistical analysis

The Mann-Whitney U test and one-way ANOVA were used to compare DT scores obtained at the time of the first cycle of chemotherapy.

Patient's OS was defined as the time between date of histological diagnosis and date of death from any cause or date of last contact/last known to be alive. Patients who were still alive at the time of analysis were censored at 31 December 2014.

Survival probabilities were estimated and expressed by Kaplan-Meier curves. Curves were compared with the log rank test. Univariable Cox proportional hazards models were used to evaluate the DT at the first cycle of chemotherapy to be a predictor for OS. In addition, univariable Cox proportional hazards models were built to evaluate the individual significance of the pretreatment covariates as a predictor of OS. Covariates (i.e., age, gender, comorbidity, histology, Charlson Comorbidity Index and (intended) treatment) were chosen as based on previous studies.

The DT score was then entered in a multivariable Cox proportional hazards model with the remaining determinants after univariable analyses. Models were used in which the DT was analyzed as a continuous variable, and as a dichotomous variable. Dichotomous variables were created by categorizing patients into two groups based on the DT cut-off value of five as proposed by Tuinman et al. [6].

P-values of p≤ 0.05 were regarded as significant. Data were analyzed with the use of IBM SPSS Statistics for Windows version 21.0.

## RESULTS

### Patients and results of the DT

Table 1 describes the included patients. Of the 495 identified patients treated with carboplatin chemotherapy, 281 were discarded from the analyses since the DT was not completed at the first cycle of chemotherapy. Of the remaining 214 patients, 113 patients were diagnosed with stage III disease. The age of these patients ranged from 37 to 79 years, with a mean of 63.3 (SD 8.7). Forty-six percent of the patients were diagnosed with adenocarcinoma. The majority of the patients received a combination of chemotherapy and radiotherapy. Thirty-nine patients (34.5%) demonstrated DT scores higher than the cut-off score of ≥ 5. No significant differences were observed between distributions of scores or mean scores of the DT for different patient characteristics.

**Table 1:** Characteristics of study population and distribution of DT scores

Characteristic	Overall sample (n=214)	Mean DT score (SD)	Median (range)	P	DT <5	DT ≥5
Age, years						
Mean (SD)	63.3 (8.7)					
Min, max	37, 79					
Sex ^a^						
Male	64 (56.6)	3.3 (2.7)	3.0 (0.0-9.0)	0.91	43 (38.1)	21 (18.6)
Female	49 (43.4)	3.3 (2.4)			31 (27.4)	18 (15.9)
DT						
Median	3.0					
range	0.0-9.0					
Histology^b^						
Adenocarcinoma	52 (46.0)	3.3 (2.5)	3.0 (0.0-9.0)	0.18	34 (30.1)	18 (15.9)
Squamous cell carcinoma NSCLC otherwise not specified, adenocarcinoma in situ	41 (36.3)6 (5.3)	3.1 (2.4)5.4 (2.9)	3.0 (0.0-8.0)6.0 (0.0-8.0)		29 (25.7)1 (0.9)	12 (10.6)5 (4.4)
SCLC	14 (12.4)	2.8 (2.9)	2.1 (0.0-9.0)		10 (8.8)	4 (3.5)
CCI						
Median	1.0					
Min, max	0, 5					
0-1 ^a^	98 (86.7)	3.2 (2.4)	3.0 (0.0-9.0)	0.42	67 (59.3)	31 (27.4)
>2	15 (13.3)	4.0 (3.3)	5.0 (0.0-9.0)		7 (6.2)	8 (7.1)
Treatment ^b^						
Chemotherapy	9 (8.0)	4.2 (2.7)	5.0 (0.0-8.0)	0.34	4 (3.5)	5 (4.4)
Surgery and (neo) adjuvant chemotherapy	14 (12.4)	3.9 (2.7)	4.0 (0.0-9.0)		9 (8.0)	5 (4.4)
Chemotherapy and sequential/concurrent Radiotherapy	90 (79.6)	3.1 (2.5)	3.0 (0.0-9.0)		61 (54.0)	29 (25.7)

Values are given in numbers (percentages) and means unless stated otherwise.

^a^ P-values calculated with the Mann-Whitney U test.

^b^ P-values calculated with one-way ANOVA. Abbreviations: n, number of patients; SD, standard deviation; DT, distress thermometer score at first cycle of chemotherapy; NSCLC, non-small cell lung carcinoma; SCLC, small cell lung carcinoma, CCI, Charlson Comorbidity Index.

### Survival estimates and cox proportional hazards models

**Figure 1:**
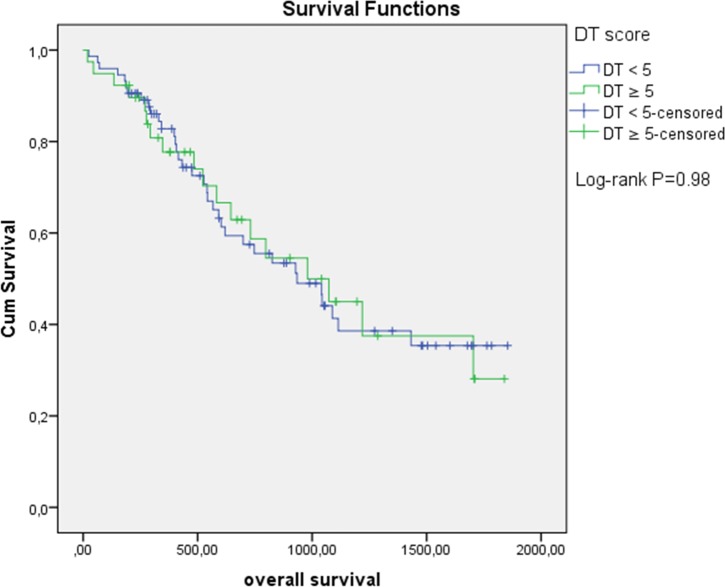
Overall survival based on Distress Thermometer (DT) score at first cycle of chemotherapy

Patients with a DT score < 5.0 did not differ to patients with a score of ≥ 5.0 with regard to age, sex, histology, comorbidity, and (intended) treatment. Figure 1 shows the Kaplan-Meier curves of the patients dichotomized by a cut-off score ≥ 5.0. No significant differences were observed (p= 0.98). Age and (intended) treatment independently predicted OS (Table 2). The DT score at the first cycle of chemotherapy as a continuous variable was in the univariable analysis not prognostic for OS. Utilizing a dichotomized DT (cut-off ≥ 5) in the univariable analysis revealed similar results. The multivariable model with age and (intended) treatment as variables demonstrated only (intended) treatment to be a significant factor for decreased OS.

**Table 2:** Results of the univariable and multivariable^a^ analyses for OS

Covariates	Univariable analyses		Multivariable analysis	
	HR	95% CI	HR	95% CI
DT	1.02	0.91-1.14		
DT				
<5	0.99	0.56-1.76		
≥5^b^				
Age	**1.04**	**1.00-1.07**	1.03	1.00-1.07
Sex				
Male Female^b^	0.71	0.41-1.22		
Histology				
Adenocarcinoma^b^				
Squamous cell carcinoma	1.72	0.95-3.13		
NSCLC otherwise not specified, adenomatous hyperplasia	1.33	0.31-5.71		
SCLC	1.01	0.43-2.39		
CCI				
0-1	0.98	0.44-2.18		
> 2^b^				
Treatment				
Surgery and (neo)adjuvant chemotherapy^b^				
Chemotherapy	1.45	0.57-3.71	1.43	0.56-3.66
Chemotherapy and sequential/concurrent radiotherapy	**6.87**	**2.16-21.85**	**6.34**	1.99-20.19

^a^All variables entered together in one block.

^b^Reference group.

Abbreviations: OS, overall survival; HR, hazard ratio; 95% CI, 95% confidence interval; DT, distress thermometer score at first cycle of chemotherapy as a continuous variable; NSCLC, non-small cell lung carcinoma; SCLC, small cell lung carcinoma, CCI, Charlson Comorbidity Index.

## DISCUSSION

To the best of our knowledge, the present study is the first in which the association between distress as measured by the DT with OS in cancer is studied. Although the DT and its problem list have some common grounds with generic HRQoL instruments, we were not able to identify it as a prognostic factor for OS in lung cancer.

It is possible that our negative results are explained by the inability of the DT to measure all aspects of HRQoL. Validity of the DT has been demonstrated by comparison with questionnaires investigating aspects related to cognitive and emotional functioning [20], but not with generic HRQoL questionnaires. Tuinman et al. demonstrated that the DT had a high correlation with the physical (r= 0.64) domain and was moderately associated with the practical (r= 0.39) and family/social domain (r= 0.31) of the problem list [6]. Generic HRQoL questionnaires, such as the EORTC QLQ-C30, address similar aspects of a patient's well-being (e.g. physical functioning/symptoms, social functioning). It would be interesting to explore whether the validity of the DT and its problem list can be established by comparing them with such instruments [6]. Similar to the study of Tuinman et al. we had to exclude identified large number of patients [6]. This may be explained by several reasons. First, patients may refuse to complete the DT and the problem list due to the length of the instrument (47 problems). Moreover, as the items of the problem list can only be answered by YES or NO, patients may not recognize their situation in these options, or may consider some of the items as irrelevant. Secondly health care personnel may not have provided the DT to patients on a regularly basis as the score of the DT would not likely result in adjustment of care. This holds true for patients considered not to experience any distress but also in patients experiencing high levels of distress in whom already extra measures are taken.

Given the previous considerations, the included patients are likely to represent a population in which patients with the best and those with the worst clinical status were not selected. It is likely this selection bias contributed to the negative results of this study. For future studies, it might be of interest to include a broader patient population and to investigate whether completion of patient outcome measures (such as the DT) is influenced by a reduced HRQoL at the start of treatment or due to treatment related side-effects.

We found the mean DT score in our patients to be comparable with other studies in lung cancer [3, 21] but lower as seen in patients with cancer from other sites [6, 7]. This finding is in contrast with the knowledge that many lung cancer patients have a bad prognosis and considerable diagnosis and treatment related stress. An explanation for this observation could be the in general low socio-economic status of lung cancer patients which could prevent them from adequately expressing their distress. Moreover, distress may also be influenced by age. Recently, it has been demonstrated that an increased age is related to the experience of decreased distress in cancer [22]. As lung cancer patients, in general, have a higher age at diagnosis, this may also explain the relatively low mean DT score. Thirdly, a considerable part of lung cancer patients have severe comorbidity (e.g. cardiac and pulmonary disease) [23] so that they are familiar with a certain amount of distress.

Given the potential relationship of the DT with global/overall HRQoL, we used only the DT without the problem list to perform the calculations in this report. However, the negative results of our study should not prevent further prospective research of the DT and the problem list beyond their intended use. The role of the DT and the problem list should be more extensively evaluated in studies investigating patient reported outcome measures to determine its concurrent validity with generic HRQoL questionnaires, to evaluate its validity and reliability in lung cancer and to assess its prognostic relevance. Such studies may offer opportunities to enhance the implementation of the DT and problem list in daily practice, to recognize patients who are prone to a negative change in HRQoL during treatment and to identify even those patients at risk for a poorer prognosis.

In conclusion, the DT was not found to be prognostic in a cohort of patients with stage III disease treated with Carboplatin. Further prospective investigations are warranted incorporating a large patient cohort with a broader treatment regimen.
